# Enabling Artificial Intelligence of Things (AIoT) Healthcare Architectures and Listing Security Issues

**DOI:** 10.1155/2022/8421434

**Published:** 2022-08-03

**Authors:** Anil Audumbar Pise, Khalid K. Almuzaini, Tariq Ahamed Ahanger, Ahmed Farouk, Kumud pant, Piyush Kumar Pareek, Stephen Jeswinde Nuagah

**Affiliations:** ^1^FinalMile Consultants Private Limited, Johannesburg, South Africa; ^2^School of Computer Science and Applied Mathematics, University of the Witwatersrand, Johannesburg, South Africa; ^3^Department of Sustainable Engineering, Saveetha School of Engineering, Saveetha Institute of Medical and Technical Sciences, Saveetha University, Saveetha Nagar, Chennai 602105, Thandalam, Tamil Nadu, India; ^4^National Center for Cybersecurity Technologies, Riyadh, Saudi Arabia; ^5^College of Computer Engineering and Sciences, Prince Sattam Bin Abdulaziz University, Al-Kharj, Saudi Arabia; ^6^Department of Computer Science, Faculty of Computers and Artificial Intelligence, South Valley University, Hurghada, Egypt; ^7^Department of Biotechnology, Graphic Era Deemed to be University, Dehradun, Uttarakhand, India; ^8^Department of Computer Science & Engineering & Head of IPR Cell, Nitte Meenakshi Institute of Technology, Bengaluru, India; ^9^Department of Electrical Engineering, Tamale Technical University, Tamale, Ghana

## Abstract

A significant study has been undertaken in the areas of health care and administration of cutting-edge artificial intelligence (AI) technologies throughout the previous decade. Healthcare professionals studied smart gadgets and other medical technologies, along with the AI-based Internet of Things (IoT) (AIoT). Connecting the two regions makes sense in terms of improving care for rural and isolated resident individuals. The healthcare industry has made tremendous strides in efficiency, affordability, and usefulness as a result of new research options and major cost reductions. This includes instructions (AIoT-based) medical advancements can be both beneficial and detrimental. While the IoT concept undoubtedly offers a number of benefits, it also poses fundamental security and privacy concerns regarding medical data. However, resource-constrained AIoT devices are vulnerable to a number of assaults, which can significantly impair their performance. Cryptographic algorithms used in the past are inadequate for safeguarding IoT-enabled networks, presenting substantial security risks. The AIoT is made up of three layers: perception, network, and application, all of which are vulnerable to security threats. These threats can be aggressive or passive in nature, and they can originate both within and outside the network. Numerous IoT security issues, including replay, sniffing, and eavesdropping, have the ability to obstruct network communication. The AIoT-H application is likely to be explored in this research article due to its potential to aid with existing and different technologies, as well as bring useful solutions to healthcare security challenges. Additionally, every day, several potential problems and inconsistencies with the AIoT-H technique have been discovered.

## 1. Introduction

**Figure 1:**
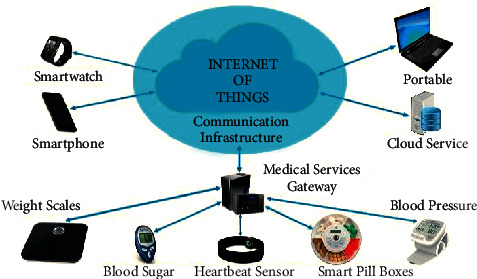
IoT-based healthcare devices [1].

AI and IoT connect physical objects and equipment by giving them the ability to see, hear, and think, which makes it possible for them to work together. They “speak” and exchange data to communicate their judgments. AIoT technologies enable previously nonintelligent devices to become intelligent by connecting objects to become intelligent by connecting them to the Internet via a variety of embedded devices, Internet protocol, sensor networks, communication protocols, and their applications. Several AIoT-based healthcare services are utilized in the medical field, such as electronic health and telecare networks, diagnosis, prevention, rehabilitation, and monitoring devices. Wireless body area networks and radio frequency recognition systems are critical components of the IoT technology, yet they are not indispensable. Although research in similar domains has exposed that remote health tracking is practicable, the possible advantages in a number of situations are substantially larger. Remote health monitoring may possibly be utilized to monitor noncritical patients from home, preferably at a hospital, lowering the demand on hospital resources, for example, surgeons and beds. This could be utilized to expand access to health care in seniors who are liberated in their homes for longer periods of time. In essence, it may increase access to clinical services, reducing the burden on healthcare institutions, and giving people more control on health over the course of time. The illustration in Figure 1 shows AIoT-based healthcare devices.

Several recent studies have focused on the IoT as a potential solution for lowering demand on healthcare systems, a widely held belief [1, 2]. A substantial portion of this study is devoted to monitoring individuals with particular diseases, for example, diabetes [3] or Parkinson's disease [4, 5]. Additional studies are being conducted to advance the range of goals, including helping patients get better by keeping an eye on their progress over time [6]. Also, emergency health care has been talked about in similar works [7] but has not gotten much attention to date.

Rehabilitation following physical damage has been the focus of numerous research studies. According to Wolgast et al. [8], this course explains the process of designing a framework that can produce a customized rehabilitation plan for a patient based on the symptoms that the patient is experiencing. In order to accomplish this, the patient's diagnosis is connected to a chart that contains the symptoms, illnesses, and treatments that have been experienced by previous patients. In 87.9% of cases, in each of the cases, the physician totally adhered to the advice provided by the system and did not make any alterations to the treatment strategy that was suggested. The current IoT technologies are evaluated in [9, 10] to determine whether or not they are suitable for use in a strategy to track Parkinson's disease patients' wearable monitors that general activity thresholds and gait patterns can be utilized in combination with vision-based sensors such as webcams in the home to notice the onset of Parkinson's disease. These technologies can work together to provide a more accurate diagnosis. In addition, the researchers hypothesized that improvements in treatment methods would one day be made possible by advances in machine learning.

The author proposed [11] a practical technique for diabetics to monitor their glucose levels in blood that procedure patient to physically acquire blood glucose levels at predetermined times. After that, the essay looks at two different types of blood glucose problems. Increased blood glucose levels are the first, while an unmeasured blood glucose level is the second. The method also chooses whom to tell, based on the severity of the irregularity: the patient, their paternity and family groups, or emergency healthcare providers like physicians. While that approach is applied and has been verified to work, automating blood glucose monitoring might improve it. Using commercially available components and a homemade antenna, the authors suggested creating a gadget for anticipating heart issues [12]. A microcontroller interprets ECG sensor data to determine cardiac rhythm. This information is subsequently sent over Bluetooth-to-user's smartphones, and then, here it is evaluated and displayed through an application. The authors observed that evolving heart attack expectation algorithms will advance the system. More gains might be made by including respiration rate monitoring, and it is said to assist in the identification of cardiac events [13].

### 1.1. Contribution to AIoT Health Care

1. Research into analogous undertakings is that no comprehensive review of AIoT accomplishments in health care has yet been undertaken
2. Comparing the effects of AIoT initiatives is to those in other healthcare businesses, as other organizations have done
3. In addition to advancing previous research, the present study provides a comprehensive analysis of the many classifications and specific components of IoT advancements in medical field
4. In keeping with the investigation's goal, this research content will be shared with stakeholders who are interested in such technological breakthroughs

The following arrangement has been determined for the remainder of this chapter.  Section 2 begins with a brief summary of artificial intelligence in health care, followed by a problem description.  Section 3 investigates the connections between the AIoT and the healthcare system. A brief paragraph in Section 4 describes the goals and major concerns in AIoT health care. This list of 5 gifts also includes AIoT equipment such as smart stethoscopes, pacemakers, and defibrillators.  Section 6 gives a synopsis of the analyses associated with this study. This section looks at how artificial intelligence (AIoT) is being used to improve medical help delivery during a crisis. The parts that follow give the conclusion and possible work.

## 2. Problem Statement

Several of the modern AIoT in healthcare publications have focused on the technology's broad uses in a range of healthcare settings, such as nursing, AAL, and surgery. Also, no clinical trials comparing the effects of AIoT advancements in health care to those of other healthcare industries have been done. As a follow-up to prior research, this paper provides a comprehensive assessment of the many sorts of specialized AIoT medical discoveries, with the determination of disseminating this info to stakeholders attracted in these kinds of developments.

## 3. AIoT and Healthcare System Interconnection

AI and IoT connect physical objects and equipment by allowing them to see, hear, and think, as well as “speak” and share information to express their decisions. AIoT technologies make previously inanimate items sentient by connecting them to the Internet via various embedded devices, communication protocols, sensor networks, Internet protocols, and applications [14]. Several AIoT-based healthcare services are employed in the medical business, including electronic health and telecare networks, diagnosis, preventive, rehabilitative, and monitoring devices. Wireless body area networks and radio frequency recognition systems are important but not key components of IoT technology. Though research in adjacent fields has shown that remote health tracking is feasible, the potential benefits in a variety of circumstances are significantly greater. Remote health monitoring could be used to track noncritical patients at home rather than in the hospital, reducing pressure on hospital services such as physicians and beds [15]. It could be utilized to assist older people stay at home for longer time by enhancing healthcare access in remote areas. In essence, it provides the ability to improve access to clinical services while simultaneously reducing pressure on healthcare facilities and giving people more control over their health. A graphical depiction of AIoT-powered healthcare equipment is shown in Figure 1 [16].

The triboelectric sensor study discussed here employed many DBN to extract attributes from raw electrical impulses from a triboelectric keyboard and perform dynamic keystroke identification [18]. However, in the absence of additional AI research, the results for gait identification employing a triboelectric sensor presented here are quite preliminary. Several strategies for automatically identifying gait types based on gait pattern measures have been proposed. A one-dimensional (1D) CNN-based technique for extracting significant characteristics from shorter segments of complete data set has been demonstrated to be particularly effective, where the feature's position within the segment is immaterial. As a result, this method works well for assessing temporal sequences of sensor data that have been divided into noisy and stable states [19].

### 3.1. AIoT for Health Insurance Companies

**Figure 2:**
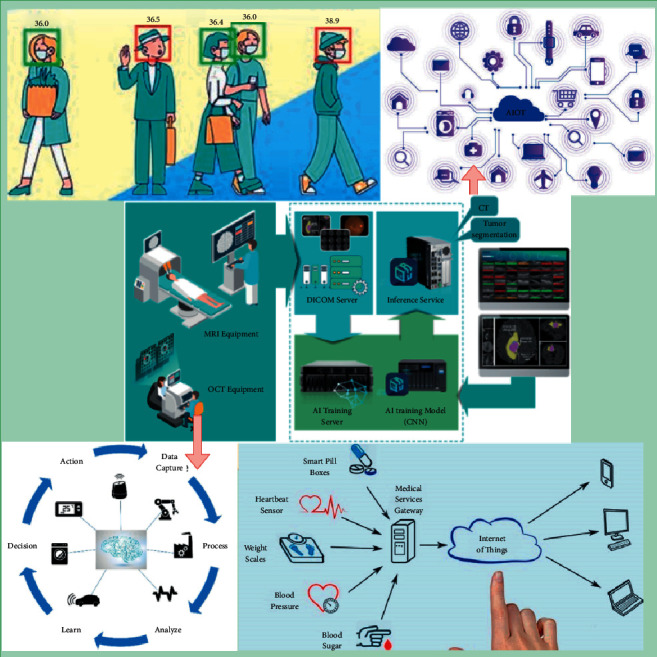
Overview of a typical AIoT-based healthcare system [17].

Healthcare devices are becoming increasingly networked, necessitating a variety of techniques to cope with the numerous scenarios that may develop as a result. Is it conceivable for insurance companies to harness data from a health-monitoring gadget to assist in insurance underwriting and operational tasks? This information will assist them in detecting and evaluating potential customers' allegations of fraud, as well as identifying those who potentially benefit from this therapy procedure. Customers might also benefit from Insurance Information Technologies (IIT). They are used not only for introducing standard underwriting and pricing but also for risk assessment [20]. Customers will be able to see the information used in each decision, supporting data-driven decisions. This enables businesses to apply in-based thinking to all elements of their operations, increasing customers' understanding of the reasoning behind every choice.  Figure 2 depicts an overview of a typical AIoT-based healthcare system.

Many insurance firms are looking into ways to reward clients for using and contributing to health data collected by AIoT devices. There are several potential techniques for improving treatment acquiescence and achieving higher levels of compliance for clients that use AIoT devices. They may provide facilities for measurable activities over which they have control. That would also aid insurance firms in their efforts to reduce liability claims. This type, like IoT data collection devices, might as well handle claims for insurance firms. It is possible that they will be able to check claims for payment from insurance companies.

### 3.2. IoT for Physicians

Rather than depending solely on health-measuring and health-monitoring devices, both are used for more accurate recording of the patient's health. Commercially accessible recordable clinical interventions can determine whether a patient is reaching their treatment goals and when they are receiving therapy. Because of the IoT, healthcare employees' growing participation in healthcare delivery has the potential for novel patterns of interactions with patients. The data from the gadgets assist doctors and nurses in making recommendations for their patients and lead to predictable results.

### 3.3. IoT for Hospitals

Many people will benefit from IoT deployment in healthcare facilities, but there will be several aspects that need more precise monitoring, such as wellness and medical diseases, which can be handled more effectively with IoT deployment. Instruments or remotely transitioning devices, such as a wheelchair, nebulizer, or blood sensor, are excellent for identifying one-time instruments since they can be monitored and measured in real time via sensors like the sensor modules. Doctor, patient, sensors, and access to cutting-edge equipment and technology like X-ray imaging may completely acquire fast, precise findings no matter how far apart they are. Patients should be informed of the risk of infection spread as it is serious. It can be used as a hygienic (safe) patient monitoring device or in conjunction with a hygienic (prophylactic) routine that decreases infection. People often use their smartphones and environmental control systems to check the temperature and humidity.

### 3.4. IoT for Patients

The IoT has altered the lives of the aged by enabling them to stay in control of their health situations all the time (24/7 service). Most importantly, it has an impact on people's lives, particularly single people and their families. When a patient is released from a hospital or returns home, a notification is issued to their family and authorized healthcare professionals, allowing them to be identified and hospitalized if they require treatment. Patients can use applications on their phones or other devices to monitor their status and keep track of their personal health and well-being using fitness bands and other wirelessly linked equipment such as blood pressure cuffs, blood pressure monitors, and glucose sensors. These gadgets are intended to calculate calories, track physical activity, test blood pressure, and do a variety of other tasks; they can be programmed to remember anything.

## 4. Objectives and Key Issues in IoT Health Care

The use of IoT in healthcare programs has the potential to result in huge advances in health care. Furthermore, it is critical to start thinking about the difference between the current structure and the future environment. In this regard, the two most crucial things are to plan the transition and to devote all of one's efforts to aiding the method in realizing its full potential. The necessity for interoperability is the first thing that springs to mind. While it is technically possible to connect all agents and computers into a single huge network, this requires common specifications and protocols. It is in everyone's best interest, as desirable, to make concessions on higher-level standards and formats that can be followed with more quality and will allow the system to improve in the future. New standardization is required to manage such a massive volume of data. Data format standardization and networking technology are required to manage huge volumes of information. The leading worry is that the “IoT” islands are kept apart from one another, which makes it difficult for them all to integrate into one single “IoT.”

The most significant hurdle to collecting comprehensive and integrated data may be the undertaking of a large transition to an interconnected network in which data from the past are blended into the current system. For a variety of reasons, data in the medical sector are extremely difficult to manage. Furthermore, patient data are complicated and/or incomplete. Its files are frequently divided geographically, and it supports a wide range of storage formats. One of the primary goals for future healthcare systems is to collect this data into meaningful repositories, create standardized databases, and make these data available to decentralized devices so that they can display and alter it in real-time or near real-time.

In this case, it is especially important to look at the IoT because it can help with the second goal that was already mentioned.

The aims on this turn are a function of the main goal, which is to construct health care that is more efficient, successful, and accessible to a bigger population. Although the structure for such an intervention already exists, the problem is determining how to proceed with the end goal of delivering healthcare facilities that maximize the possibilities of technology. When thinking about change management, the philosophy behind change management comes to mind. As shown in the table above, various institutional transformation models are available, ranging from low risk/reward (up) to high risk/reward (down), as well as in terms of health care and the IoTs. This idea extends to businesses in the example given, as demonstrated by the move to a system that employs networked data and devices to facilitate the flow of healthcare data to all providers [21].

## 5. AIoT Healthcare Devices

The IoT is being used in healthcare initiatives. The impact of limited healthcare resources on the laboratory technician workforce, as well as fewer healthcare professionals as a result of the placement of health-related IoT devices, has the potential to decrease the burden of blood shortage. Doctors can treat infections and track patient well-being while also supporting therapies using invasive, transdermal medication to pacer systems, electronic gauges, and further drug monitoring. However, expansion has various advantages as well as countless threats. Many people have brought up these clear concerns about healthcare protection. Information acquired and saved by AIoT embedded devices can be accessed by data thieves. For widespread adoption, the use of Internet of Healthcare devices and networks would need to be enhanced much further. Cybersecurity is a significant barrier to AIoT usage in health care.

### 5.1. Blood Coagulation Testing

A Bluetooth-enabled coagulation device has been released by Roche. Patients can utilize the AIoT system to measure the pace at which the blood clots. Roche's AIoT platform is the first designed particularly for anticoagulated patients. Self-testing patients are less likely to bleed or have a stroke since they keep within their treatment range. Fewer patient visits were required due to the ability to electronically communicate test findings to their healthcare practitioner. In addition, Roche's device allows patients to make notes on their test findings, tells patients to test again, and emphasizes test findings that are outside of a particular range.

### 5.2. Connected Inhalers

Asthma is a significant medical illness that affects lots of people all over the world. People with asthma who use inhalers can raise their reservoir, providing them with more control over their symptoms and treatment as they have state-of-the-art asthma software. Propeller has created a sensor, which will be able to control an inhaler/spirometer from a distance. A sensor is set up to notify patients with asthma and COPD about their thoughts and also facts that may aid them in making choices about their health. Sensors and software are used to notice medicine use and allergen presence, as well as to predict and alert customers about these changes. Some people thought the attached inhaler had significant advantages because it required more effort on the part of the patient to use. The sensor also produces a report that can be referred to by the patient's doctor.

### 5.3. Glucose Monitoring

**Figure 3:**
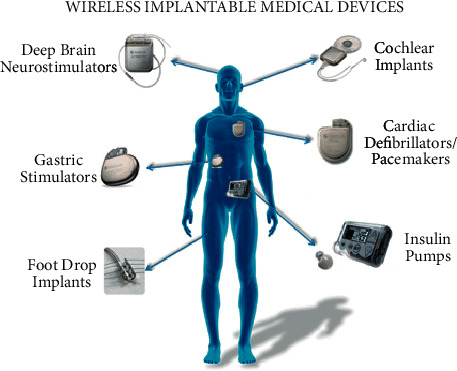
Wireless implantable medical devices [1].

For more than 30 million diabetic Americans, glucose control has always been a concern. It takes time to manually monitor and document glucose levels, and it only notes a patient's glucose levels during the test. If levels change dramatically, routine monitoring is insufficient to detect a difficulty. IoT solutions that allow incessant, automated glucose monitoring of patients may help to relieve these concerns. Patients are notified when their blood glucose levels are irregular by glucose monitoring devices, which reduces the need for manual record keeping. Wireless implantable devices based on AIoT are depicted in Figure 3. Developing an AIoT system for glucose monitoring is one of the problems:

1. Small enough to be utilized unobtrusively to track over time without causing inconvenience for patients
2. It does not consume so much energy that it needs to be recharged on a regular basis

These are not insurmountable problems, and devices that resolve them could change how people with diabetes control their blood sugar levels.

### 5.4. Bluetooth-Enabled Blood Laboratories

The SFIT, Lausanne, has developed implantable laboratories that allow for independent examination of patients' blood samples, giving crucial information. The implant is made up of five electrodes, each of which has an enzyme coating and can detect substances like glucose and lactate. The item is scanned, and Bluetooth is activated on the individual's PC when it is discovered. More research may be necessary. However, using a mobile connection, the results of this inquiry might be sent to a doctor in another place for evaluation. By reducing reliance on physicians, this great discovery would mostly assist the elderly and chronically ill. An implantable device removes the necessity for additional blood tests once it has been implanted, resulting in a decrease in laboratory personnel. An automated laboratory that removes the need for face-to-face interaction with patients reduces time spent gathering all essential laboratory tests, which helps the already overburdened healthcare system in a number of ways.

### 5.5. Connected Cancer Treatment

The findings of a clinical study including patients with head and neck cancer were presented at the 2018 ASCO Annual Meeting. As in this pilot study, all of the patients wore a Bluetooth device while getting care. Their pain, heart rate, and weight were collected through Bluetooth and constantly monitored by a blood pressure and pulse-tracking program. Each patient's weight was also counted every day to give their doctor important information. The drug may be modified on a daily basis if the doctor deems it necessary. Participants in the trial showed diminished symptoms when compared to those in the placebo group who had never received any medical facilities related to cancer or individuals receiving medical facilities one time a week.

Technology has aided in the simplification of patients' treatment by supporting them with any developing side effects, and also labeling and alleviating their apprehensions, as well as enabling the recognition of effects caused by the implementation of advanced healthcare practices and the implementation of smart health practices. Smart technology therapies may lessen patients' issues and annoyance. It was found that more contact between patients and doctors makes health care better and cuts down on the time people spend in doctors' offices. This means that more people can do more everyday things [22].

### 5.6. Robotic Surgery

By inserting tiny Internet-connected robots within the human body, surgeons can perform complex operations that would not be possible with human hands. Simultaneously, robotic procedures performed by small AIoT devices would significantly decrease the size of incisions required for surgery. This will make the treatment less unpleasant and allow patients to recover more quickly.

### 5.7. IoT-Connected Contact Lenses

Lens enlargement and lens stiffening treatments like extracapsular cataract extraction are intended to finally cure the cataract ophthal eye condition known as acquired long needs. A research project aims to address long-lens failure, also known as presbyopia, which causes long-fickleness. In order to ensure recovery, it will examine lens healing, refracting, and stiffening. Swiss researchers developed Sensimed signals, which use noninvasive equipment to detect changes in ocular pressure that could suggest glaucoma.

### 5.8. A Depression-Monitoring Smartwatch App

Patients can utilize a tracking system on a regular basis to measure their emotions and their condition for MDD. This is to wear a smartwatch. There is a clear chance for wearable technology to have a greater impact than step counting in this circumstance; gadgets that assess the intensity of depression will be appropriate. Like other IoT apps, a depression app could give patients and their caregivers more data about their problems.

### 5.9. Connected Wearables

Without linked wearables, the IoT looks incomplete. Since worn and linked sensors are important instruments for medical staff, it helps as well as assists patients. They allow health practitioners to monitor, for example, heart rates, body temperatures, pulses, and additional key body metrics while still working to assess people's health. Furthermore, hospitals have the advantage of wearing devices with continuous contact, which allows physicians to track patients long after they have left the clinic. It is especially helpful in assisting patients with recurring checkup appointments if they discuss any issues they encountered in the hospital after discharge. If the condition varies, the wearable sensors would notify the doctor from anywhere. If a doctor receives a real-time warning, they can deliver a live-time direction to their patients.

## 6. Associated Research's Summary

Intelligent healthcare systems are an essential component of smart city development. Promotional actions are focused on the establishment of a “digital health” system. Using the smart city cloud platform, the smart city would create a medical health large data platform that will allow information exchange and exchange among medical and health organizations, and also a citizen's medical health large data center that will encourage three-way medicine linkage and classified identification treatments. Additionally, citizens will have access to electronic health records, enabling them to access the network of health services offered by hospitals and clinics all over the area. Encourage the city to adopt online registration, electronic toll collection, remote telemedicine service, diagnostic technology for visual and physical examinations, and other medical and health-related advancements. The current study looked into a number of aspects of the AIoT system. This article examines the architecture, components, and communication between them of an AIoT system in detail. This article also addresses existing healthcare services for which IoT-based solutions have been researched. Using these ideas, IoTs have assisted healthcare experts in monitoring and diagnosing a number of health situations, measuring a variety of health indicators, and offering diagnostic services in inaccessible places. That has moved the emphasis of the healthcare industry from hospital-based to patient-based initiatives. In addition, we emphasized the numerous applications and current advances of AIoT technology. The difficulties and issues associated with the AIoT plan for the system and manufacture, and there is also been talk about utilities. Those challenges would serve as the foundation for future development and study goals. There is also thorough, up-to-date information about AIoT devices for those who want to learn more about the topic or advance their knowledge. The AIoT's quick growth is because of its major advantages of greater accuracy, lower costs, and the ability to suggest upcoming happenings. Regardless of the fact that smartphone and computer technologies have long been outdated, the growing accessibility of applications and IoT, as well as wireless technology and virtual economy, have all added to the fast AIoT, leading society's whole technological ecosystem to continue to develop [21].

Additional physical instruments (sensors, actuators, and so on) have been combined with AIoT products (which may be equipped with sensors, actuators, and so on) to collect and share data using protocols such as Bluetooth, Wi-Fi, and IEEE 802.11 [23]. To integrate clinical details about the patient, heart-related applications use embedded or wearable sensors, for example, a thermistor, a palpatory, a lead electrophysiologist's graph, and an electro-measurement pathologist of the electric potential of the brain. Temperature, humidity, and time of day are all examples of environmental factors that can be recorded. These medical data can be used to draw intriguing and particular inferences about the patients' clinical status. As IoT sensors collect and capture diverse information from the Internet, as well as the accumulation of large volumes of data, it is offered by a diversity of causes (mobile phones, software, sensors, e-mail, and applications). The information acquired by the preceding study is distributed to physicians, carers, and anyone authorized to access devices. The expansion of cloud/server-style diagnostics and dissemination of these details with healthcare services is a well-organized use of data. Treatments are administered immediately if necessary. At the moment, the solutions available focus on sensors and smart refrigerators. Temperature monitoring has long been mandated by storage safety regulations. Temperature monitoring is not a new idea, and with today's technological advancements and IoT solutions, data from refrigerators can be monitored, saved in the cloud, and analyzed. People who use the app, the healthcare institution, and contact module operate simultaneously, so everyone can use the app data. Human-interface components are used in the bulk of IoT (in the system, AIoT serves as a console for medical consultants, allowing for patient monitoring, data visualization, and apprehension). The study found that AIoT in health care has enhanced the findings of the preceding year by investigating additional difficulties. Healthcare surveillance, regulations, and privacy are the possible AIoT applications that must be researched initially. Previously mentioned technological achievements show how successful and profitable the AIoT has the potential to be in the healthcare industries. The main problem, though, is keeping quality of service matrices that promote data exchanges, stability, costs, and resilience while still keeping each user's information private [24].

In Table 1, we examined the contributions of a variety of researchers to AIoT in health care. In 2019, Dang et al. devised a cloud-based technique for AIoT analysis in health care [24]. They chose to address a number of security-related and cloud computing implementation-related issues and concerns in health care. In 2019, a significant number of peer-reviewed journal paper reviews were released [26, 26–29], and in 2018 [25, 30], both of these works sought publication prior to 2017. Nazir et al. conducted an exhaustive analysis of research published between 2011 and 2019 that labels the security and privacy risks of mobile-based healthcare IoT [29]. There are currently no studies devoted exclusively to the application of AIoT in medicine. However, research works on AIoT in health care are presented in Table 1.

Table 2 is a summary of the findings of 10 studies (such as systemic analyses and other types of reviews) that examined the application of AIoT in health care. The first study [20] was a thorough AIoT review of health care that examined networks and designs, methods, and implementations. They discussed numerous themes, including protection and standards, AIoT and e-Health legislation and policies, and standardization and protection. By contrast, the content in this paper was written, while AIoT was just beginning. Because AIoT is gaining importance in the healthcare industry, a new study is required.

In 2018, a comprehensive analysis of the materials and innovations utilized in healthcare AIoT implementations was provided in [30] (see Table 1). According to the survey, the home is the most prevalent location for healthcare AIoT. The top-ranked journals for articles on the topic are Procedia Computer Science, Journal of Network and Computer Applications, and Journal of Medical Systems, according to the data. Another 2017 [31] study showed that the hospital was the most vulnerable setting for AIoT installation. Definite countries' e-health programs and policies have been mentioned as working on AIoT deprived of giving a description of their efforts [20, 26]. Additional problem that AIoT raises is security and confidentiality, also interoperability and integration with technology issues in health care. We have discussed these topics in greater depth in previous papers [18, 20, 21, 25, 27, 29, 32–34]. Previously, these scholarly publications concentrated on various areas of AIoT health care, for example, application of cloud computing, fog computing, mobile computing, wearable sensors, and big data in health care. Numerous study journals on the existing state and prospective advances for healthcare AIoT analyze the conditions and future developments.

## 7. Applications of IoT to Build Emergency Medical Services

**Figure 4:**
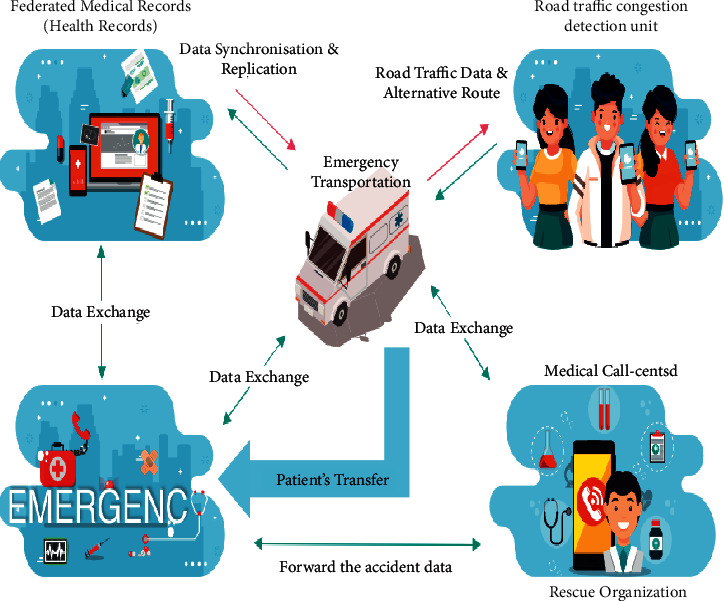
Important areas of IoT analytics in medicine [36].

A reference model for IoT applications has been developed with the help of academics. In fact, Jin et al. [35] give a framework for project planning in their research article “Creating an IoT Implementation for Smart Cities.” According to the article, the IoT will help to the development of a smart city in three ways: IoT data-centric, cloud-centric, and network-centric. These viewpoints serve as a reference model for creating smart cities as a result of considering many of the different uses of smart development initiatives.  Figure 4 depicts some crucial areas of AIoT analytics in medicine.

### 7.1. Data-Centric IoT

When considering that perspective, attention is on information, as a big volume of data produced by IoT devices would result in a massive volume of data. The term “data-centric IoT” refers to the concept of all aspects of data flow, including data analysis, transmission, storage, and display.

#### 7.1.1. Collection of Data

Data collection is the initial phase in data flow, and it has a significant influence on following phases such as network access, data storage, energy usage, and system architecture. The data gathering approach can be a combination of random sampling and continuous sampling when the infrastructure is either stationary or mobile. A more modern sensing technique was devised and deployed, based on RFID and WSN, but a new one has emerged: participatory sensing. It achieves this by leveraging the profusion of sensor-rich, Internet-enabled cellphones, as well as the population's mobility, to enable hitherto unattainable finer, more granular sensing. Extending participatory sensing technologies has numerous benefits, including zero hardware investment and on-demand sensing. However, the basic question of whether participatory sensing will produce accurate data remains unanswered. A different way to get more people involved would be to use a variety of incentives and competitions.

#### 7.1.2. Management and Preprocessing of the Dataset

After obtaining raw data, it must be processed in order to provide valuable information and intelligence. When it comes to this phase, it is usually a good idea to do some preprocessing and event detection, with the latter accomplished by using event detection from long-duration time series data. To handle data from a wide range of temporal and spatial dimensions, algorithms must be adaptive and robust. To provide a complete interpretation of the results, complex computational approaches, for example, evolutionary algorithms, neural networks, and genetic algorithms, should be utilized to convert raw data into knowledge. Data retention would still be just as important as it is now, and letting data be used to keep historical documents safe and do research on them as time goes on.

#### 7.1.3. Interpretation of the Dataset

The final objective of data-centric IoT design is to transform raw sensor data into data that produce insight and knowledge. Modern computer hardware and digital new technologies make things easier to do, making knowledge display more intuitive and accessible to users. Touch screen displays are among the most contemporary technologies available, allowing users to connect to and navigate data through the use of a touch screen. 3D displays improve data visualization by reflecting data in a more accurate manner [37].

### 7.2. Cloud-Centric IoT Platform

**Figure 5:**
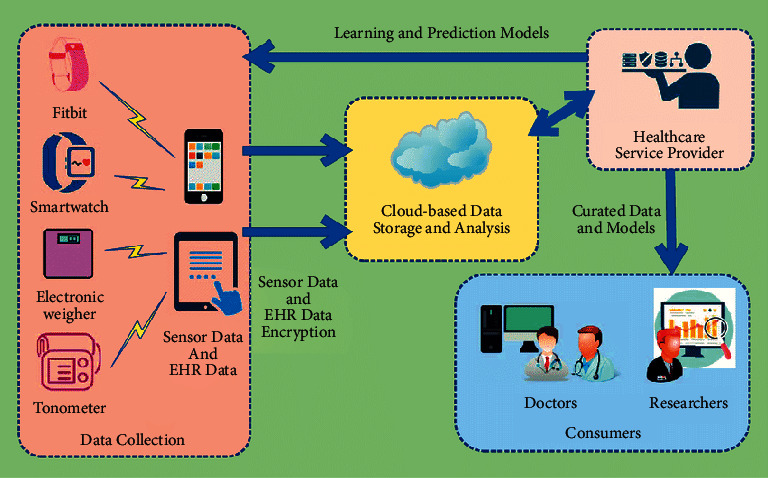
Systematic overview of the cloud-centric IoT platform [38].

The cloud-centric IoT architecture, which fully utilizes the possibilities of cloud computing, is designed to include IoT features and smart city technologies while harnessing the benefits of cloud computing. In this system, sensors connected to network generate data that are subsequently kept in cloud storage with the help of software engineers producing framework-supporting software. Also, data mining and deep learning specialists are employed to convert raw data into facts and intelligible language. There are numerous cloud computing services available, comprising IaaS, PaaS, and SaaS. Therefore, data acquired by sensors, software that maintains the device, and algorithms that interpret the data would be shielded from public access. A cloud-based IoT architecture will combine every parts of distributed calculating and offer storage and processing resources that can be scaled up or down.  Figure 5 depicts a pictorial representation of a systematic overview of a cloud-centric IoT platform.

### 7.3. Network-Centric IoT

Either an Internet-based or object-based network-centric IoT architecture can be used. The Internet will be the major priority for data transfer in the Internet-based system, with the Internet serving as a key component of the architecture. Smart objects can be used to create object-oriented designs. In this network-centric AIoT approach, the networking infrastructure serves as the network kernel. Like older architectures, network-centric IoT designs put a lot of weight on different types of sensors, addressing devices, networking models, and QoS processes.

#### 7.3.1. Sensing Paradigm

One of the prevalent sensing paradigms is WSN, RFID, and crowd sourcing. Items and activities are labelled with RFID tags. When the symbols are questioned by the reader, they respond with their unique IDs, which are subsequently relayed to the reader. Given the rising usage of RFID technology in the transportation and access control industries, the application of RFID in a smart city is natural. WSN is major demonstrative of the sensing paradigm, and it consists of four important activities: collecting valuable information from external areas; evaluating the data; decoding the data; and transmitting the results. Sensor features such as enhanced power, compactness, and dependability, as well as decreased production costs, are now widely used. Sensor networks, for example, are growing more significant as sensors become smaller, more dependable, and more powerful. Crowd sourcing, a current sensing approach, is a participative Internet-enabled system that is interested in everyone's devices. By upgrading our crowd sensing technology, we are able to provide sensing capabilities that were previously unavailable due to their limited temporal and spatial granularity. Future devices will have even more sensors as cellphones can already see more of the world and how many people are in it.

#### 7.3.2. Addressing Scheme

In this strategy, we focus on giving each artifact a unique identification. This enables all instruments to be recognized and differentiated, as well as connected to and monitored. We would design the network in such a way that the increased number of devices and networks would not impair its performance. IPv4 has yet to show signs of vulnerability and is still widely used on the Internet. The address space, on the other hand, would be nearly empty in no time. The new Internet Protocol version 6 is a worthy successor to IPv4, with a substantially bigger address space that ensures unique addresses for all computers on the planet. Furthermore, IPv6 contains characteristics that function across a wide range of formats and networking networks, but it is still simple to use on devices that do not have access to them. In a network-centric AIoT design, the Zigbee protocol provides another strong addressing mechanism for AIoT devices. Each Zigbee device has three distinct addresses: a 16-bit network address, a permanent 64-bit MAC address, and a string of names used for various purposes [39]. Each Zigbee machine has its own 64-bit MAC address, although the network address on Zigbee network is 16 bits and is established by the Zigbee network supervisor or router. This method of addressing is perfect for the complication and routing needs of Zigbee schemes.

#### 7.3.3. Connectivity Model

The TCP/IP architecture was essential in building the foundation for the Internet. Because TCP/IP layers are integrated into a layered configuration, WSNs use this architecture to meet their own standards, such as low-power service, network scalability, and minimal resources. The original TCP/IP architecture had several networking layers, with every tier contributing resources to the bottom layer. The OSI model has five layers: the physical layer, the MAC layer, the network layer, the transport layer, and the application layer. WSN operations employ the same architecture.

#### 7.3.4. Quality of Service (QoS) Mechanism

An IoT design includes a variety of heterogeneous networking networks, network operation, and quality of service due to the diversity of protocols (wired and wireless) and sensor kinds. Like the Padova Smart City Project has numerous AIoT applications, each with its own set of characteristics, as well as traffic, delay requirements, network types, and power sources. This is an excellent illustration of an IoT architecture's diversified structure. To better understand this term, we consider two types of network traffic: elastic throughput and elastic delay tolerant traffic, and inelastic bandwidth and inelastic delay tolerant traffic.

With quick growth of wearable biosensors and wireless communication technology, several smart healthcare solutions for real-time patient health monitoring are now available. These systems, however, have numerous security problems. A password guessing attack, for example, can negotiation AIoT devices, resulting in an attack of health data privacy. Following an introduction of the security challenges related with healthcare AIoT, this paper investigates the security weaknesses involved with password creation and suggests a technique for evaluating password strength that uses users' personal data into consideration. The following section goes over the security and privacy concerns for AIOT health care.

## 8. Security and Privacy Requirements of AIoT Health Care

**Figure 6:**
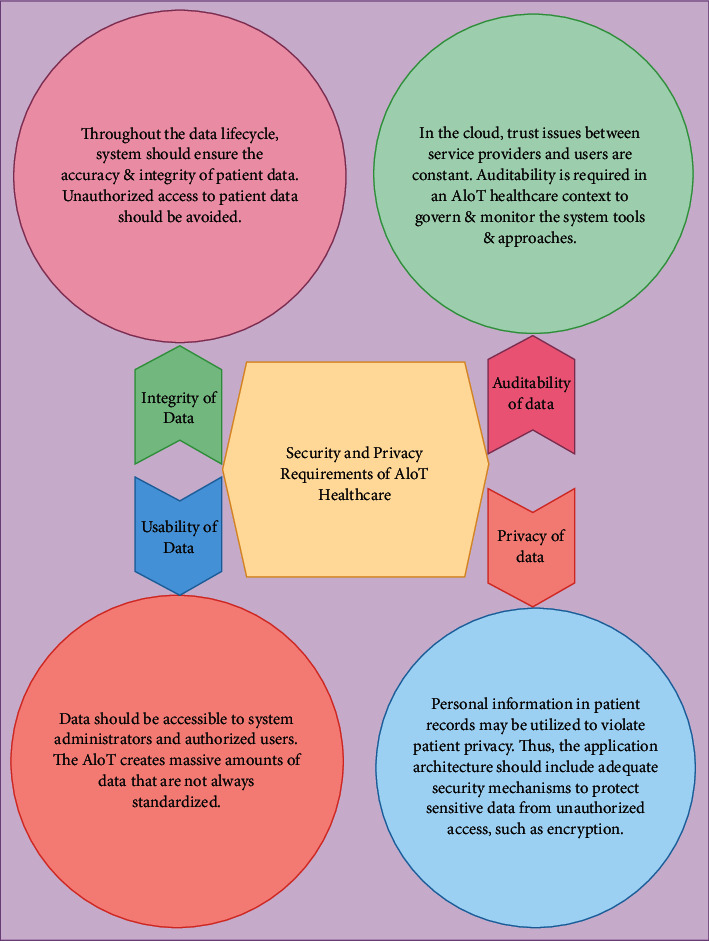
Security and privacy requirements of AIoT health care [31].

Clinical diagnostics and emergency medical response to patients in medical care facilities and at home via remote medical procedures are among the numerous healthcare services and uses. Patients' information is maintained in a cloud-based data center or database. Furthermore, the cloud provides access to numerous healthcare specialists, such as those that conduct medical diagnoses in order to treat patients. Traditional ways of taking care of patients are being changed quickly by these facilities, making medical care more effective and efficient [40]. Furthermore, these healthcare applications allow for remote monitoring through smart devices like smartphones and wearable sensor devices. Academics interested in the issue may want to investigate a number of prospective AIoT-H implementations to gain a better knowledge of the impact and influence on a variety of AIoT applications. In Figure 6, a diagrammatic presentation of the AIoT Healthcare Security and Privacy Requirements is shown.

The key purposes of a cloud data center are data management, data security protection, and data storage. Data storage functions include structured data storage, video data storage, image data storage, and semistructured data storage. The primary concerns of data management are data indexing, data fusion, data analysis, and data visualization. It is important to encrypt and separate sensitive data, as well as control access, manage permissions, make backups, restore data, and keep audit logs [40, 41].

IoT healthcare apps must be safe and secure in order to enhance patients' health. They must also consider the security and privacy threats that they bring to patients, as well as other issues, for example, privacy violations and financial hazards [42]. By digging into the components of the application architecture, this chapter examines privacy and security concerns associated with IoT healthcare apps.

Cellphones and wearable sensor devices with remote monitoring capability are two examples. Healthcare services and software are used to assist remote patient monitoring processes and alert systems. Because the data produced by these procedures are deemed sensitive, it is the most valuable asset of these apps. This is because it has a direct impact on the health and well-being of patients. The insinuations of cooperating sensitive data are all times serious, with a major impact on the overall system's and its stakeholders' privacy and security. Furthermore, the data's confidentiality and availability must be safeguarded. Sensitive data should be secure against illegal access as well as other dangers and concerns. Therefore, these are difficult zones in an AIoT ecosystem because volume of data generated by sensor devices and the continual connectivity among the devices in system. In latest days, healthcare experts and app creators have worked to create secure AIoT healthcare apps to address these tasks [43].

## 9. Conclusion and Future Work

One of its aims of e-Health is to deliver healthcare assistance to individuals at home, notably through the use of AIoT. AIoT applications are often designed to save money while also inspiring patients in their homes, getting more patient involvement. This will result in better health promotion and a more fulfilling lifestyle for everyone. Based on this study's results, IoT in the medical field is still in its early stages. The utilization-limiting application appears to have placed a major burden on the healthcare system in a number of subfields. Moreover, as the number of medical AIoT studies and research domains expanded in 2018, so did the number of studies on this topic, culminating in the future inclusion of new study fields.

Patients, healthcare experts, and insurance firms are all connected in healthcare industry, which is a large and complex enterprise. Regardless of the fact that AIoT is not yet widely used in a range of medical sectors, it is gaining traction in those fields. Regardless of the fact AIoT, healthcare, and medicine have a lot in common, there are some areas where AIoT has yet to be utilized due to interdisciplinary challenges. This investigation may be of interest to academics who are especially interested in understanding this phenomenon. This aim will be achieved in the upcoming time both qualitatively through interviews with besieged interviews or statistically through literature surveys.

## Figures and Tables

**Table 1:** General review of IoT in health care.

Paper title	Contributions	Author and year
The IoT for health care: a comprehensive survey	(i) Discuss the issues and concerns surrounding the IoT in health care (ii) We will discuss several rules and regulations governing the IoT and eHealth. We will talk about big data, ambient knowledge, and wearables in health care (iii) in IoT for health care, we investigate network topologies and accompanying software or facilities	Riazul Islam et al. [20] 2015

Medical IoT and big data in healthcare	(i) Investigate the application of IoT and big data in health care	Dimitrov [21] 2016
	(ii) Discuss the issues around the usage of big data in health care	
	(iii) Apps and smartphone applications are being tested	

IoT for smart health care: technologies, challenges, and opportunities	(i) A definition of IoT use in health care is proposed	Baker et al. [23] 2017
	(ii) The existing state of affairs as well as potential future developments in healthcare IoT are discussed	
	(iii) Discuss prospective research trends, problems, and challenges related to IoT in health care	
	(iv) Consider cloud computing to be a type of data storage system	
	(v) Pay attention to various wearable and networking devices	

Advanced IoT for personalized healthcare system: a survey	(i) Examine and classify healthcare IoT systems, deployments, and case studies	Yang et al. [24] 2017
	(ii) Present a four-tier IoT architecture for specialized healthcare networks (PHS)	
	(iii) Discuss upcoming advancements in research, as well as issues and roadblocks in healthcare IoT	
	(iv) Present a summary of the current state and projected future advances of healthcare IoT	

Toward fog-driven IoT eHealth: promises and challenges of IoT in medicine and health care	(i) Specify a multitiered architecture for the IoT-enabled e-Health ecosystem: utilize devices, fog computing, and a cloud service to inspire	Farahani et al. [25] 2018
	(ii) Discuss the IoT's impact on hospitals and pharmacies	
	(iii) Present a unified platform for the IoT-enabled eHealth environment	

**Table 2:** Systematic review of IoT in health care.

Paper title	Contributions	Author and year
A survey on IoT and cloud computing for healthcare	(i) Focus on architecture, platforms, and topologies when you study the IoT framework for the healthcare sector	Dang et al. [26] 2019
	(ii) Assess the impact of the IoT and cloud computing on health care	
	(iii) Describe new IoT and cloud computing trends and projects in the healthcare industry around the world	
	(iv) Talk about how the IoT and cloud computing in health care affect security	
	(v) Consider the issues posed by the usage of IoT and cloud computing in health care	

The application of IoT in health care: a systematic literature review and classification	(i) Investigate the healthcare IoT's cybersecurity and interoperability issues	Ahmadi et al. [30] 2019
	(ii) Show the current state of IoT in health care, followed by potential future changes	
	(iii) More information regarding the IoT and how it could be incorporated into health care	
	(iv) Examine the benefits of cloud-based IoT healthcare infrastructure	

IoT for health care using effects of mobile computing: a systematic literature review	(i) Show the current state of IoT in health care, followed by potential future changes	Nasir et al. [29] 2019
	(ii) Analyze the security and privacy concerns brought by IoT in health care	
	(iii) IoT and mobile computing in health care	

IoT-based healthcare applications: a review	(i) This shows the current state of IoT in health care, followed by potential future changes	Itamir de and Gibeon [27] 2019
	(ii) Examine the security and privacy risks posed by the IoT in health care	
	(iii) IoT and mobile computing in health care	

Enabling technologies for fog computing in healthcare IoT systems	(i) The current state and potential future developments in healthcare IoT are discussed	Mutlag et al. [28] 2019
	(ii) Collect their functional and nonfunctional needs	
	(iii) Discuss the difficulties and questions around healthcare IoT infrastructure	

## Data Availability

The data that support the findings of this study are available on request to the corresponding author.
